# Prevalence and structural correlates of HIV and STI testing among a community-based cohort of women sex workers in Vancouver Canada

**DOI:** 10.1371/journal.pone.0283729

**Published:** 2023-03-30

**Authors:** Shira M. Goldenberg, Jennie Pearson, Sarah Moreheart, Hannah Nazaroff, Andrea Krüsi, Melissa Braschel, Brittany Bingham, Kate Shannon

**Affiliations:** 1 Division of Epidemiology and Biostatistics, School of Public Health, San Diego State University, San Diego, CA, United States of America; 2 Centre for Gender and Sexual Health Equity, Vancouver, BC, Canada; 3 Division of Social Medicine, Department of Medicine, University of British Columbia, Vancouver, BC, Canada; 4 Faculty of Health Sciences, Simon Fraser University, Burnaby, BC Canada; 5 Vancouver Coastal Health, Indigenous Health, Vancouver, BC, Canada; Uniformed Services University: Uniformed Services University of the Health Sciences, UNITED STATES

## Abstract

**Background:**

In light of the stark inequities in HIV and sexually transmitted infections (STIs) experienced by women sex workers, empirical evidence is needed to inform accessible and sex worker-friendly models of voluntary, confidential and non-coercive HIV and STI testing. We evaluated the prevalence and structural correlates of HIV/STI testing in the last 6 months in a large, community-based cohort of women sex workers in Vancouver, Canada.

**Methods:**

Data were drawn from an open community-based open cohort of women sex workers (January 2010-August 2021) working across diverse street, indoor, and online environments in Vancouver, Canada. Using questionnaire data collected by experiential (sex workers) and community-based staff, we measured prevalence and used bivariate and multivariable logistic regression to model correlates of recent HIV/STI testing at enrollment.

**Results:**

Of 897 participants, 37.2% (n = 334) identified as Indigenous, 31.4% as Women of Color/Black (n = 282), and 31.3% (n = 281) as White. At enrollment, 45.5% (n = 408) reported HIV testing, 44.9% (n = 403) reported STI testing, 32.6% (n = 292) reported receiving both HIV and STI testing, and 57.9% (n = 519) had received an HIV and/or STI test in the last 6 months. In adjusted multivariable analysis, women accessing sex worker-led/specific services had higher odds of recent HIV/STI testing, (Adjusted Odds Ratio (AOR): 1.91, 95% Confidence Interval (CI): 1.33–2.75), whereas Women of Color and Black women (AOR: 0.52, 95%CI: 0.28–0.98) faced significantly lower odds of recent HIV/STI testing.

**Conclusions:**

Scaling-up community-based, sex worker-led and tailored services is recommended to enhance voluntary, confidential, and safe access to integrated HIV/STI testing, particularly for Women of Color and Black Women. Culturally safe, multilingual HIV/STI testing services and broader efforts to address systemic racism within and beyond the health system are needed to reduce inequities and promote safe engagement in services for racialized sex workers.

## Introduction

Sex workers face significant health and social inequities related to criminalization, occupational violence, human rights abuses, and a disproportionate burden of HIV and sexually transmitted infections (STIs) compared to other populations [1–4]. Access to regular testing represents an essential component of comprehensive HIV and STI prevention and influences entry into the ‘cascade’ of HIV and STI treatment and care. Access to safe, voluntary, and appropriately tailored testing services is especially important given the asymptomatic nature and health sequelae of many STIs [5]–for example, the potential for untreated STIs to exacerbate vulnerability to HIV acquisition. In comparison with substantial efforts to scale-up services along the HIV cascade of care, attention to STI testing has been more limited. The World Health Organization notes that “despite the availability of several simple, cost-effective interventions to combat STIs, little progress has been made” in STI prevention and control [6]. Additionally, while progress has been made on scaling-up access to HIV testing and the care cascade for many populations, sex workers represent a key population that continues to be disproportionately left behind in these efforts [1, 7, 8].

Given the stark HIV and STI inequities experienced by sex workers, empirical evidence is needed to inform accessible and sex worker-friendly models of voluntary, confidential and non-coercive HIV and STI testing. Previous literature has examined individual and health-system factors associated with HIV and STI testing among sex workers, such as substance use, cost, and provider stigma [8]; however, a growing body of evidence speaks to the importance of criminalization, policing, gender-based violence, and other structural factors that may impede access and engagement with HIV/STI prevention, treatment, and care [1, 2, 8, 9]. Despite high need for safe and accessible sexual health and HIV care, sex workers face high levels of discrimination, criminalization, socio-economic marginalization, and occupational stigma. These structural factors have been shown to pose barriers to quality and timely healthcare for sex workers globally [10–17] and in Canada [18–20]. However, they have been infrequently explored in relation to HIV and STI testing access and outcomes. At the same time, community mobilization interventions such as sex worker-led and tailored outreach, drop-in spaces, and other services (e.g., advocacy, anti-stigma campaigns) have been shown to confer significant reductions in HIV and STI incidence and risks among sex workers [21–24], yet have been infrequently examined in relation to HIV/STI testing utilization. Finally, despite general evidence of racial and im/migration-related disparities in HIV/STI services utilization [25–27], few epidemiologic studies have investigated racial and im/migration-related inequities in HIV/STI testing among women sex workers globally. This information remains critical for advancing evidence-based responses to systemic racism influencing access and engagement with health care. This is particularly vital in the Canadian context, where there is a need for research and interventions to address longstanding anti-Indigenous and anti-Black racism within and beyond the health system [28–30], as well as the ongoing exclusion of precarious and racialized im/migrants from healthcare [25, 30, 31].

In light of known barriers to health access faced by sex workers generally, and the individual and public health importance of regular HIV/STI testing, our objective was to evaluate the prevalence and structural correlates of recent (last six months) HIV/STI testing at enrollment in a diverse, community-based cohort of women sex workers in Canada. Based on previous literature, we hypothesized that marginalized and racialized subgroups (e.g., trans women, Indigenous women, women of color, im/migrants) would experience lower uptake of testing, whereas engagement with sex worker-led and tailored services would be associated with increased uptake.

## Methods

### Study design

Data for this analysis were drawn from an ongoing, prospective, open cohort, An Evaluation of Sex Workers Health Access (AESHA), which initiated recruitment in 2010. AESHA was developed based on collaborations with sex work agencies since 2005 [32], is monitored by a Community Advisory Board of partner organizations and community members, and has included experiential staff (current/former sex workers) since inception. The study received ethical approval from the University of British Columbia/Providence Health Care Research Ethics Board. Written informed consent was obtained from all participants prior to enrolment in the study. A waiver of the requirement of parental consent was waived by the Research Ethics Board for participants <age 18 under the emancipated minors clause.

Eligibility includes identifying as a cis or trans woman, 14 years old or older at enrolment, exchanged sex for money within the last 30 days, and providing written informed consent. Our recruitment criteria are inclusive of diverse and fluid identities while capturing the ways that patriarchal gender norms and cissexism shape participants’ experiences in sex work. Eligibility is inclusive of cis women, transgender women, transexual women and other self-reported transfeminine identities at enrolment. To address the challenges of recruiting stigmatized and hidden populations such as sex workers, time-location sampling was used to recruit participants through daytime and late-night (9 pm–2 am) outreach to outdoor/public sex work locations (e.g., streets, alleys) and indoor sex work venues (e.g., massage parlors, micro-brothels, and out-call locations) across Metro Vancouver, BC. In addition, online recruitment was used to reach sex workers working through online solicitation spaces. Indoor sex work venues and outdoor solicitation spaces (‘strolls’) are identified through community mapping and updated regularly by the outreach team. The response rate was 85%, with the primary reason for non-participation being not actively engaged in sex work in the past 30 days. As an open cohort, recruitment remains ongoing to refresh and replenish the cohort on an annual basis to maintain an overall sample size of N~800.

Following informed consent, sex workers completed interviewer-administered questionnaires at baseline and semi-annually. Interviews were conducted at study offices in Metro Vancouver or a confidential space of participants’ preference (e.g., home, work). The questionnaire is administered by a trained interviewer and data are securely collected and managed using REDCap electronic data capture tools [33, 34]. The questionnaire included questions related to individual socio-demographic characteristics, sexual and drug risk exposures, and structural factors including work environment, criminalization, and diagnoses and access to health services, including HIV and STI testing, treatment, and care, as well as primary care, mental health, sexual and reproductive care, and Hepatitis C. The questionnaire is updated regularly to capture emerging and changing priorities and needs within the community. Participants received $60 Canadian at each study visit for their time, expertise and travel. At each study visit, following extensive pre-test counselling, participants were offered voluntary HIV/STI/HCV serology testing by a project nurse and offered referrals or STI treatment onsite, if needed. All participants received post-test counselling.

### Outcome

Building on previous AESHA research [24], this analysis sought to measure prevalence and correlates of HIV/STI testing at enrollment. The outcome for this analysis was a binary (Yes/No) variable measuring if a participant had received HIV or STI testing within the six months prior to their baseline visit. Testing utilization was assessed separately for HIV and STIs and combined for the purposes of analysis. Because cohort follow-up involves voluntary onsite HIV/STI testing visits, this analysis was restricted to baseline data (collected at enrollment).

### Independent variables

Socio-demographic variables included measures of *racialization and Indigenous identity* to examine the effects of racism, defined as Indigenous (inclusive of First Nations, Métis, or Inuit), Women of Color (e.g., East Asian, Southeast Asian), Black women, and white. Given the low proportion of Black participants, Black and Women of Color were combined for analysis to examine experiences of racialized identities. Other socio-demographic variables included *age* (measured continuously, in years, and categorized as younger than 30 years old vs. 30+), education (high school attainment vs. less than high school), *im/migration to Canada*, *sexual minority* (gay, lesbian, bisexual, asexual, queer vs. heterosexual), *gender identity* (including trans women, transexual women and other nonbinary identities vs cisgender women), and history of *mental health diagnoses*.

Variables capturing experiences in the last six months included sexual and drug risks, including: *inconsistent condom use with clients* (defined as using condoms less than 100% of the time for vaginal or anal sex with regular or occasional clients), *non-injection* and *injection drug use* (excluding alcohol and cannabis), and whether participants had *exchanged sex for drugs in the last 6 months*. Structural factors capturing experiences in the last six months included *unstable housing*, assessed by asking “in which of the following types of places have you slept overnight in the last 6 months’; *negative police encounters while working*, based on the question, “have you experienced any of the following encounters with police in the last 6 months?”; *incarceration*, measured as ‘yes’ to the question, “in the last 6 months, have you been in detention, prison or jail overnight or longer for any reason at all?”; and *primary place of solicitation*, assessed by asking “In which of the following ways have you solicited/hooked up with your clients?” Responses were coded as *outdoor/public* (e.g., street, park), *indoor* (e.g., bar, nightclub, massage/beauty parlor, micro-brothel), or *independent (e*.*g*., escort agency, online, phone). *Use of sexual and reproductive health services* was assessed by asking “Have you ever used any of the following health and support services?”. Responses that included local sexual and reproductive health services were coded as yes, whereas those who did not select any of these responses or a valid ‘other’ response were coded as no. *Use of sex worker-specific/led services* was assessed by asking “Have you ever used any of the following health and support services?”. Responses of mobile outreach, drop-in spaces, or other community-based services led by or tailored to sex workers were coded as yes.

### Statistical analyses

Descriptive statistics for individual and structural characteristics were calculated as frequencies and proportions for categorical variables and measures of central tendencies (i.e., median, interquartile range [IQR)) for continuous variables. These were stratified by the outcome and compared using Pearson’s chi-square test for categorical variables (or Fisher’s exact test for small cell counts), and the Wilcoxon rank-sum test for continuous variables.

Bivariate and multivariable analyses used logistic regression to examine associations with recent HIV/STI testing. Structural exposures of interest (e.g., criminalization, use of outreach services, racialization) and individual confounders hypothesized to be associated with recent HIV/STI testing were included in the theoretical multivariable model. Statistical analyses were performed in SAS version 9.4 (SAS, Cary, NC), and all p-values are two-sided. Missing data were handled using a complete case approach.

## Results

Analyses included 897 participants enrolled from January 2010 –August 2021. At enrollment, 45.5% (n = 408) had accessed HIV testing, 44.9% (n = 403) STI testing, 32.6% (n = 292) both HIV and STI testing, and 57.9% (n = 519) HIV and/or STI testing in the last 6 months. Of those who recently tested, 73.7% (297/403) successfully received their STI test results and 90.7% (370/408) received their HIV test results. Among those who received STI test results, the most common modes of receiving results were in-person (61.6%), with few receiving results by phone (17.2%), through outreach (7.1%), or online (<1%). Primary STI testing sites accessed included family doctor offices and private walk-in clinics (22.6%), low-barrier community clinics (51.1%), and mobile testing services (8.4%) (e.g., mobile van, street nurses). Similar sites were accessed for HIV testing; private walk-in clinics (16.2%), low-barrier community clinics (58.3%), and mobile testing services (9.1%).

The median age of participants was 35 years (IQR: 28–42), just over half of the sample had high school or higher educational attainment (n = 494, 55.1%), and the proportion who used non-injection or injection drugs was 65.9% and 39.8%, respectively (Table 1). With regards to racialization, 1.6% (n = 14) were Black, 29.9% (n = 268) were Women of Color (Asian and Southeast Asian), 37.2% (n = 334) were of Indigenous ancestry (inclusive of First Nations, Inuit, and Metis), and 31.3% (n = 281) were white; 29.9% of women were im/migrants to Canada from other countries, with China representing the primary country of origin, with significantly lower proportions of HIV/STI testing among Black/People of Color as well as among im/migrants. Primary places of soliciting clients included outdoor/public space (47.4%), indoor (30.1%), and independent (e.g., online) (21.0%), 35.9% had experienced negative police encounters while working, and 13.5% had been in jail or prison overnight or longer in the last 6 months. With regards to health and social supports, 58.8% (n = 527) and 25.9% (n = 232) of participants had accessed sex worker-led/tailored services and sexual and reproductive health services in the last 6 months, respectively, both of which were much more common among those accessing testing.

**Table 1:** Baseline individual and structural factors stratified by recent HIV/STI testing (last 6 months) at enrollment among women sex workers in Metro Vancouver, Canada (n = 897) AESHA, 2010–2021.

Characteristic	Total (%) (*n* = 897)	Outcome		*p—*value	
		Recent HIV/STI testing* (n %) n = 519	No recent HIV/STI testing* (n %) n = 378		Missing data (n)
Age (median, IQR)	35 (28–42)	33 (27–41)	36 (30–44)	<0.001	
Younger than 30 years old	282 (31.4)	191 (36.8)	91 (24.1)	<0.001	
Sexual Minority†	335 (37.4)	237 (45.7)	98 (25.9)	<0.001	8
Gender Minority***	76 (8.5)	55 (10.6)	21 (5.6)	0.007	3
High school attainment	494 (55.1)	260 (50.1)	234 (61.9)	<0.001	
Mental health diagnosis	437 (48.7)	295 (56.8)	142 (37.6)	<0.001	4
Non-injection drug use*	591 (65.9)	408 (78.6)	183 (48.4)	<0.001	
Injection drug use *	357 (39.8)	262 (50.5)	95 (25.1)	<0.001	
Unstable housing*	637 (71.0)	429 (82.7)	208 (55.0)	<0.001	4
*Racialization*					
White	281 (31.3)	188 (36.2)	93 (24.6)		
Indigenous	334 (37.2)	244 (47.0)	90 (23.8)		
Women of Color and Black Women**	282 (31.4)	87 (16.8)	195 (51.6)	<0.001	
Im/migrant to Canada	268 (29.9)	83 (16.0)	185 (48.9)	<0.001	
Primary place soliciting clients*					14
Outdoor or public space	425 (47.4)	304 (58.6)	121 (32.0)		
Indoor	270 (30.1)	85 (16.4)	185 (48.9)		
Independent	188 (21.0)	121 (23.3)	67 (17.7)	<0.001	
Inconsistent condom use with clients*	162 (18.1)	115 (22.2)	47 (12.4)	<0.001	6
Exchanged sex for drugs*	260 (29.0)	181 (34.9)	79 (20.9)	<0.001	13
Negative police encounter while working *	322 (35.9)	215 (41.4)	107 (28.3)	<0.001	
Incarceration*	121 (13.5)	91 (17.5)	30 (7.9)	<0.001	10
Accessed sex worker-led/ tailored services*	527 (58.8)	374 (72.1)	153 (40.5)	<0.001	
Accessed sexual and reproductive health services*	232 (25.9)	171 (33.0)	61 (16.1)	<0.001	

† Gay, lesbian, bisexual, two-spirit, asexual, queer, or other

* In the last six months

** Women of Color and Black Women were considered as a combined variable due to small sample size considerations and the importance of being able to consider racialized identities.

***Gender minority included all trans and non-binary identities, including trans women, transexual women and other nonbinary identities vs cisgender women

In bivariate analysis, structural factors which were positively and significantly associated with higher odds of recent HIV/STI testing included Indigenous ancestry and accessing sex worker-led/tailored services (Table 2); Black/Women of Color and im/migrant women had reduced odds of testing. In multivariable analysis adjusted for confounders, the odds of recent HIV/STI testing were higher among women accessing sex worker-led/specific services (adjusted odds ratio (AOR): 1.91, 95% confidence interval (CI): 1.33–2.75), and lower among Women of Color and Black participants (AOR: 0.52, 95% CI: 0.28–0.98) (Table 2). Sensitivity analyses examining STI testing separately yielded similar findings to the combined models, including increased testing among sex workers who accessed outreach services (AOR: 2.26, 95% CI: 1.55–3.28), and reduced odds among im/migrant sex workers (AOR: 0.34, 95% CI: 0.23–0.50).

**Table 2:** Bivariate and multivariable analysis of factors correlated with recent STI/HIV testing (last 6 months) at enrollment among sex workers in Metro Vancouver, Canada (n = 897), AESHA 2010–2021.

	Unadjusted	Adjusted
Characteristic	Odds Ratio (95% CI)	Odds Ratio (95% CI)
Youth	1.84 (1.37–2.47)	1.53 (1.09–2.13)
Sexual Minority†	2.44 (1.83–3.25)	1.57 (1.12–2.20)
Gender Minority***	2.02 (1.20–3.40)	0.93 (0.52–1.69)
*Racialization*		
Indigenous	1.34 (0.95–1.90)	1.19 (0.82–1.71)
(vs. white)		
Women of Color and Black Women**	0.22 (0.16–0.32)	0.52 (0.28–0.98)
(vs. white)		
Im/migrant to Canada	0.20 (0.15–0.27)	0.76 (0.41–1.44)
Inconsistent condom use with clients*	2.00 (1.38–2.90)	1.23 (0.81–1.89)
Injection drug use*	3.04 (2.27–4.06)	1.33 (0.92–1.91)
Non-injection drug use*	3.92 (2.93–5.24)	1.09 (0.67–1.75)
Exchanged sex for drugs*	1.99 (1.46–2.71)	0.82 (0.56–1.20)
Accessed sex worker-led/ specific services*	3.79 (2.86–5.02)	1.91 (1.33–2.75)

† Gay, lesbian, bisexual, two-spirit, asexual, queer, or other

* In the last six months

** Women of Color and Black Women were considered as a combined variable due to small sample size considerations and the importance of being able to consider racialized identities.

***Gender minority included all trans and non-binary identities, including trans women, transexual women and other nonbinary identities vs cisgender women

## Discussion

In this prospective, community-based cohort study of women sex workers, accessing community-based, sex worker-tailored and/or led services (e.g., outreach, drop-in spaces) was associated with higher odds of HIV/STI testing, whereas racialized Women of Color and Black Women faced disparities in HIV/STI testing. Findings suggest the need to scale-up community-based, sex worker-tailored services to enhance voluntary, confidential, and safe access to integrated HIV/STI testing for sex workers. Culturally safe, multilingual services and broader efforts to address systemic racism within and beyond the health system are urgently needed to reduce inequities and support testing access for racialized sex workers.

In this study, women accessing sex worker-led/tailored services (e.g., outreach, community-based drop-in spaces) had almost two-fold higher odds of utilizing HIV/STI testing.

These findings add to a growing body of evidence regarding the importance of community empowerment interventions in facilitating access to HIV/STI prevention among sex workers globally [21, 23, 35]. A global systematic review showed community empowerment interventions to be associated with reductions in HIV (odds ratio [OR]: 0.68; 95% confidence interval [CI]: 0.52–0.89), gonorrhea (OR: 0.61; 95% CI: 0.46, 0.82), chlamydia (OR: 0.74; 95% CI: 0.57, 0.98), and high-titre syphilis (OR: 0.53; 95% CI: 0.41, 0.69) and increased consistent condom use with clients (OR: 3.27; 95% CI: 2.32, 4.62) [21]. Our study adds to this body of research by highlighting the association between sex worker-led community outreach and drop-in services and improved access to HIV/STI testing, which is supported by previous research suggesting similar effects on linkage to sexual and reproductive health care in this setting [36].

Despite these encouraging findings, we identified important gaps in sex workers receiving testing results, with 24% of those receiving STI testing and 4% of those who utilized HIV testing not having received their results, respectively. Enhanced efforts are needed to provide sex workers with testing results and to ensure linkage to voluntary, non-stigmatizing, and appropriate HIV and STI treatment and care services [37]. As sex workers experiencing structural barriers to health services require different approaches from those used by traditional public health follow-up systems [1, 20, 38, 39], strategies for testing follow-up and linkage to care that are supported and led by sex workers and sex worker-allied organizations may facilitate improved access and outcomes [1, 22]. Additionally, adopting and integrating evaluated rapid point-of-care tests for STIs [40]—alongside already approved HIV point-of-care testing—represents an opportunity to ensure individuals receive results promptly, affording more occasion for treatment and follow-up. Finally, structural interventions that address criminalization are urgently needed to enhance the pervasive criminalization of both sex work and HIV non-disclosure. Criminalization fuels stigma [19, 41] and can undermine sex workers’ engagement in HIV and STI testing services [1, 2, 9, 41], as concern regarding potential negative criminal and social consequences of testing may overshadow personal health concerns. Decriminalization of sex work [42, 43] and the removal of overly broad approaches to HIV criminalization [44, 45] and punitive public health interventions [46] are urgently needed evidence-based interventions.

In this study, Women of Color and Black participants faced 48% reduced odds of recent HIV/STI testing compared to white women; this study represents one of few to have examined race-based disparities in testing among sex workers, and is consistent with U.S. and Canadian research documenting important racial inequities in HIV prevention engagement and outcomes (e.g., testing, PreP) among other key populations, such as men who have sex with men [47, 48], and adolescent girls [49]. These results add to a body of evidence regarding the harms of systemic racism on inequities in health care among Black, Women of Color, and im/migrants [28, 30, 50, 51]. Our findings are supported by a global systematic review that found experiences of racism to be associated with more negative patient experiences of health services, including healthcare-related trust, satisfaction, and communication, as well as delays or avoidance of care [52]. Findings suggest the need for future research and interventions to address racial inequities in HIV/STI testing among Black and Women of Color sex workers, including through supporting community-led HIV and sex work organizations which are comprised of and support Black and Women of Color. Systemic efforts to recognize, measure, and dismantle systemic racism within and beyond the health system are needed to support access to safe and inclusive HIV/STI testing, prevention, and care services for Black and Women of Color, whose needs remain sorely neglected in the Canadian context and elsewhere[50, 53, 54].

We found that im/migrant women faced reduced odds of HIV/STI testing compared to their Canadian-born counterparts, although this was not statistically significant after adjustment for racialization. Nonetheless, bivariate findings echo previous work indicating that racialized im/migrant women in sex work face structural barriers to healthcare access resulting from language barriers, criminalization, police surveillance, and occupational stigma [55, 56]. Im/migrant sex workers in Vancouver are over-represented in managed indoor establishments [57], which often face restrictions on the promotion of HIV/STI prevention services under sex work laws in Canada which criminalize many third party activities (e.g., advertising, material benefits) [55, 57]. For temporary permit holders, there are additional prohibitions on working in businesses related to the sex trade [58], which further exacerbate barriers to HIV/STI prevention [59]. Research has shown that im/migrant sex workers often fear that accessing sexual health services or disclosing their work to healthcare providers could result in negative impacts on their workplace (e.g., police raids) or ability to work [55, 57, 56, 60]. Finally, recent research suggests im/migrant workers are less likely to engage in sex worker community organizing and sex work-specific services [24], which have been linked to improved sexual health outcomes [24].

### Strengths and limitations

Strengths of this study include its strong community collaborations, and large, diverse sample. As with most research involving stigmatized populations, there is potential for social desirability and recall bias in self-reported behavioral measures; our community-based and experiential team, training in non-stigmatizing interview techniques, and community collaborations are designed to mitigate such bias. This research cannot infer causality and findings may not be fully generalizable to other sex worker populations, including Black women, who may be underrepresented. However, the mapping of working areas and time–location sampling likely enabled us recruit a large and diverse sample of sex workers across varied work environments, minimizing potential selection bias. Our community-based approach has also been previously shown to be critical for mitigating potential reporting bias in research with marginalized populations. Finally, although HIV and STI testing are often evaluated and delivered separately, this study focused on both forms of testing, given that such needs often co-occur among sex workers, and that these services may be most effectively delivered through integrated delivery models [61–63]. Unfortunately, certain descriptive measures–for example, modality for receiving test results—was only asked of participants receiving STI testing.

## Conclusions

In this community-based cohort, women accessing sex worker-led services had higher odds of recent HIV/STI testing, while Women of Color and Black women faced substantial inequities in access. Findings suggest the need to scale-up community-based, sex worker-tailored services to enhance voluntary, confidential, and safe access to integrated HIV/STI testing for sex workers. Culturally safe, multilingual services and broader efforts to address systemic racism within and beyond the health system are urgently needed to reduce inequities and improve testing access for im/migrant and racialized sex workers. Future research should focus on ways to ensure that HIV/STI testing results and appropriate treatment are communicated and provided, as well as to design and evaluate interventions for increasing access to safe, voluntary, and non-stigmatizing testing services.
